# Thermodynamic investigation of the interaction between ionic liquid functionalized gold nanoparticles and human serum albumin for selective determination of glutamine†

**DOI:** 10.1039/d0ra04394j

**Published:** 2020-08-26

**Authors:** Sushama Sahu, Reshma, Srishti Sharma, Indrapal Karbhal, Kallol K. Ghosh

**Affiliations:** School of Studies in Chemistry, Pt. Ravishankar Shukla University Raipur-492010 C.G. India kallolkghosh@gmail.com +91-771-2262583

## Abstract

The excellent biocompatible and monodispersed gold nanoparticles (AuNPs) functionalized by amino based ionic liquid (IL) have been synthesized for the demonstration of their interaction with human serum albumin (HSA). Amino based IL stabilizes the surface of AuNPs and provides a colorimetric sensor platform. The size of synthesized IL–AuNPs was identified by use of transmission electron microscopy (TEM) and dynamic light scattering (DLS) techniques. Molecular interaction of functionalized AuNPs with HSA have been investigated using multispectroscopic techniques, such as UV-Vis, fluorescence and Fourier transform infra-red (FT-IR) spectroscopy. The fluorescence and synchronous fluorescent intensity together indicated that IL–AuNPs exhibits a strong ability to quench the intrinsic fluorescence of HSA *via* a dynamic quenching mechanism. Moreover, the binding constant (*K*_a_), Stern–Volmer quenching constant (*K*_SV_) and different thermodynamic parameters, namely Gibb's free energy (Δ*G*), enthalpy (Δ*H*) and entropy (Δ*S*) have been evaluated at different temperatures. This interactive study focuses on the nature of surface modification of IL–AuNPs *via* HSA for selective detection of glutamine (Glu) with a lower limit of detection of 0.67 nM in the linear range of 10–100 nM for Glu.

## Abstract 2

The excellent biocompatible and monodispersed gold nanoparticles (AuNPs) functionalized by amino based ionic liquid (IL) have been synthesized for the demonstration of their interaction with human serum albumin (HSA).

## Graphical abstract

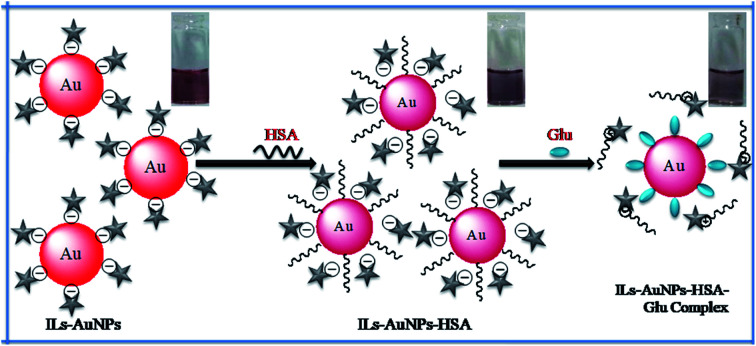

## 1. Introduction

Serum albumins are proteins found in blood plasma which account for about 60% of the total protein corresponding to a concentration of 42 g L^−1^ and are ultimately responsible for about 80% of the osmotic pressure of blood.^1–3^ Both bovine serum albumin (BSA) and human serum albumin (HSA) are thiol containing proteins.^4^ HSA consists of 585 amino acids and the main binding sites are located at hydrophobic cavities in their subdomains, such as tryptophan (Trp-214) and tyrosine (Tyr-411) residues.^5^ HSA has a tendency to interact with positively charged species due to its negative charges at pH 7.8 and the isoelectronic point (pI) of HSA is found to be 4.9, hence it shows roll on distribution, transportation and metabolism functions in pharmaceuticals.^5,6^ HSA is considered as biodegradable and non-antigenic, hence, it is widely used for the preparation of microsphere and nanosphere sized nanoparticles (NPs).^7^

Understanding the interaction of NPs with serum albumin is an important aspect in nanobiology, nanomedicine and nanotoxicology. The assembled HSA-NPs exhibits enhanced permeability and retention effect (EPR effect), hence, it enables too passive tumor targeting in cancer therapy.^8^ Additionally, albumin molecules possess many functional groups which helps them in binding to the surface of other bio-active ligands, such as different amino acids found in human blood.^7,9^ Hence, it is necessary to study the interaction ability of HSA towards NPs as they may be a key for many bio-medical issues.

From past decades, NPs have been targeted as a novel and innovative nanomaterials due to their unique properties and potential applications with high chemical reactivity.^8^ There nano size enables them to interact with other biomolecules, drugs *etc.* making them applicable in areas such as catalysis or microelectronics.^10^ Metal NPs are extensively used in therapeutic and diagnosis due to their excellent properties, such as minimal size, high stability, large surface area, suspension reactivity and tunable water solubility.^11^ Mostly, metal NPs include gold (Au), silver (Ag), copper (Cu) and iron (Fe). Among these, Au has been studied extensively in many fields, as optical absorption study, self assembled monolayers, immunoassay and resonance light scattering spectroscopy, due to their inert and relatively less cytotoxicity.^12^ Nowadays, ionic liquid (IL) are also becoming a subject of interest for the functionalization of metal NPs due to their extraordinary physical, chemical and biodegradable properties.^13^ Therefore, the study of the interaction of IL–AuNPs to serum albumins for selective sensing of amino acids could be critically significant.

A lot of research work has been focused in understanding the mechanism behind interaction of NPs with serum albumins.^4,7,13–15^ The major interaction for NPs–HSA binding involves π–π stacking, electrostatic and hydrophobic interactions.^16^ The nature and sources of the interaction of NPs with HSA also contribute in unraveling the binding mechanism between them.^7^ Hemmateenejad *et al.*^17^ investigated the interaction of ZnS-NPs with HSA and indicated that fluorescence of HSA is quenched through static mechanism. They also showed the spontaneous binding reaction and proved that conformational structure of HSA molecules could be changed in the presence of ZnS-NPs using synchronous fluorescence spectroscopy. Sen *et al.*^18^ studied the interaction of AuNPs with HSA by surface energy transfer method and proved that AuNPs interacts more strongly with subdomain IA of HSA. Gorjup *et al.*^19^ developed and optimized the ligand modified NPs based on HSA for an efficient gene therapy. Chamani *et al.*^20^ studied the interaction between ciprofloxacin and HSA in the presence and absence of AgNPs to describe the critical aggregation concentration changes of ciprofloxacin by changing the hydrophobic interaction in presence of AgNPs. So far, no work has been reported involving the study of interaction between AuNPs functionalized by amino based IL with HSA for selective detection of amino acids.

**Fig. 1:**
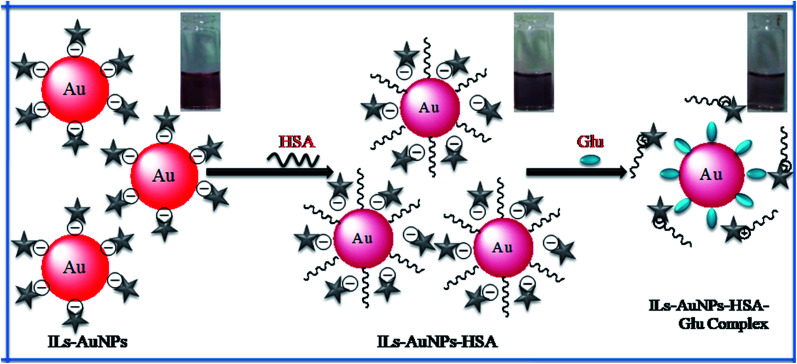
Schematic representation for the interaction of IL–AuNPs with HSA for selective sensing of Glu.

Here, in this work, fluorescence and synchronous quenching method as well as UV-Vis method have been used for the determination of different parameters to evaluate the interaction between IL functionalized AuNPs with HSA. Based on the HSA quenched by IL–AuNPs, different parameters as Stern–Volmer quenching constant (*K*_SV_), enthalpy (Δ*H*), entropy (Δ*S*) and Gibb's free energy (Δ*G*) were calculated by fluorescence and UV-Vis technique at different temperature. This interaction study has been applied to investigate their effect for the detection of amino acids found in human blood. This interaction strategy has been applied for selective detection of glutamine (Glu) using IL–AuNPs as a colorimetric sensor. In this method, the amino based ionic liquid (IL), *i.e.*, 4-((hydroxyimino)methyl)-1-(2-(octylamino)-2-oxoethyl)pyridin-1-iumbromide [Fig. S1(a)†] was used for functionalization of AuNPs surface for obtaining strong absorption peak in the visible region. The self aggregation behavior of AuNPs has been reduced due to the use of IL. The synthesized IL–AuNPs in the absence and presence of HSA was characterized by UV-Vis, fluorescence, transmission electron microscope (TEM), dynamic light scattering (DLS) and diffused reflectance Fourier transform infra-red (DRS-FTIR) spectroscopy. The aggregation of IL–AuNPs was driven upon the addition of HSA which causes decrease in absorption band at 520 nm whereas fluorescence (FL) intensity is found to be decreased by increasing the concentration of IL–AuNPs. Further, this interaction was applied for selective sensing of Glu, in which all HSA molecules were found to be replaced by amino and carboxyl group of Glu [Fig. S1(b)†] confirmed by strong FL intensity observed for AuNPs–Glu complex. The schematic representation of this process is shown in Fig. 1.

## 2. Experimental section

### 2.1 Materials

All the chemicals used were of analytical grade. Hydrogen tetrachloro aurate(iii) (HAuCl_4_·3H_2_O, 99%), human serum albumin (HSA), glutamine (Glu), aspartic acid (Asp), leucine (Leu), trypsine (Try), tyrosine (Tyr), arginine (Arg) and methionine (Met) were procured from Sigma Aldrich Pvt. Ltd., Bangalore, India. HSA was dissolved in 0.1 M phosphate-buffer saline (PBS), pH 7.8 and stored at 4 °C. Sodium borohydride (NaBH_4_) was purchased from Merck, Mumbai, India. Amino based ionic liquid (IL) *i.e.*, 4-((hydroxyimino) methyl)-1-(2-(octylamino)-2-oxoethyl) pyridin-1-iumbromide was purchased from Sigma Aldrich Pvt. Ltd. Bangalore, India. All the chemicals were used without further purification. All the experiments were performed with ultrapure water (18 MΩ cm).

### 2.2 Instrumentation

The concentration and interparticle distance of IL–AuNPs and IL–AuNPs with HSA were measured from 200 nm to 800 nm (pH 7.8) at 291, 298 and 305 K on Agilent technology Cary-60 UV-Vis spectrophotometer. Fluorescence measurements were performed on a Fluorescence spectrophotometer equipped with a 150 W xenon lamp in the wavelength range of 300–450 nm using Agilent technology Cary eclipse fluorescence spectrophotometer. Here, the excitation wavelength was 280 nm and scan rate was 600 nm min^−1^. The excitation and emission slits were taken to 5.0 nm for both. Fourier transform infra-red (FT-IR) spectra of AuNPs, IL–AuNPs and IL–AuNPs with HSA were taken *via* diffused reflectance method performed on a Nicolet iS10 FT-IR (Thermofisher) using KBr matrix in the range of 500 to 4000 cm^−1^ at room temperature. Transmission electron microscope (TEM) measurements were performed on a JEOL, JEM-2100F, operated at accelerating voltage 200 kV to identify the size and shape of IL–AuNPs and IL–AuNPs with HSA. Dynamic light scattering (DLS) measurement was performed in nano-zetasizer instrument (Malvern, UK) to determine the size distribution by intensity and zeta potential of the sample solution.

### 2.3 Synthesis of AuNPs functionalized with IL

The monodisperse IL functionalized AuNPs were synthesized by reduction of hydrogen tetrachloro aurate with sodium borohydride according to the previous work with slight modification.^21–23^ Briefly, IL–AuNPs were synthesized by reduction of hydrogen tetrachloro aurate(iii) in the presence of NaBH_4_ as reducing agent. For this, 0.25 mL of IL was added into a 25.0 mL hydrogen tetrachloro aurate(iii) solution (2.0 × 10^−3^ M) in conical flask with constant stirring. After 15 min, 0.25 mL of NaBH_4_ (2.0 × 10^−3^ M) was added dropwise into the sample solution under vigorous stirring at room temperature. After 10 min, the color of solution mixture changes to wine red indicating the formation of IL–AuNPs. This solution was stirred for another half an hour to ensure that IL self assembles onto the surface of AuNPs. The concentration of synthesized IL–AuNPs has been found to be 9.3 × 10^−6^ M using the light absorption method.^24^

### 2.4 Procedure for HSA interaction studies with IL–AuNPs

An aliquot of HSA (0.3 mL, 1 × 10^−5^ M) was added in glass vial containing 2.0 mL of IL–AuNPs and the total volume of the solution mixture was making up to 3.0 mL with ultrapure water. Fluorescence and UV-Vis spectrophotometer were used to monitor the FL intensity and absorbance, respectively for HSA before and after the addition of IL-AuNPs. Fluorescence and synchronous fluorescence measurement was used to determine the binding and Stern–Volmer quenching constants (*K*_SV_) by the plot of quencher (IL–AuNPs) concentration *versus* fluorescence (FL) intensity in HSA solution. UV-Vis spectra were also drawn between concentrations of IL–AuNPs in the range of 3.1 × 10^−5^ M to 3.1 × 10^−4^ M. Absorbance and the linear fit plot could be used to determine the different thermodynamic parameters for the complex of IL–AuNPs–HSA.

### 2.5 Sensing procedure of glutamine using IL–AuNPs–HSA complex

This interaction phenomenon has been employed for selective sensing of Glu in the presence of other kind of amino acids abundantly found in human blood. For that, same concentration of the entire amino acids (0.3 mL, 100 nM) has been added to 3.1 × 10^−6^ M IL–AuNPs–HSA sample solution by making up the solution 3.0 mL and then investigated *via* spectrophotometric technique. This study was demonstrated at room temperature with 10 min of reaction time. The concentration of amino acid has applied for quantification of the FL-intensity ratio for IL–AuNPs–HSA complex before and after the addition of amino acids.

## 3. Results and discussion

### 3.1 Characterization of IL–AuNPs and IL–AuNPs with HSA

**Fig. 2:**
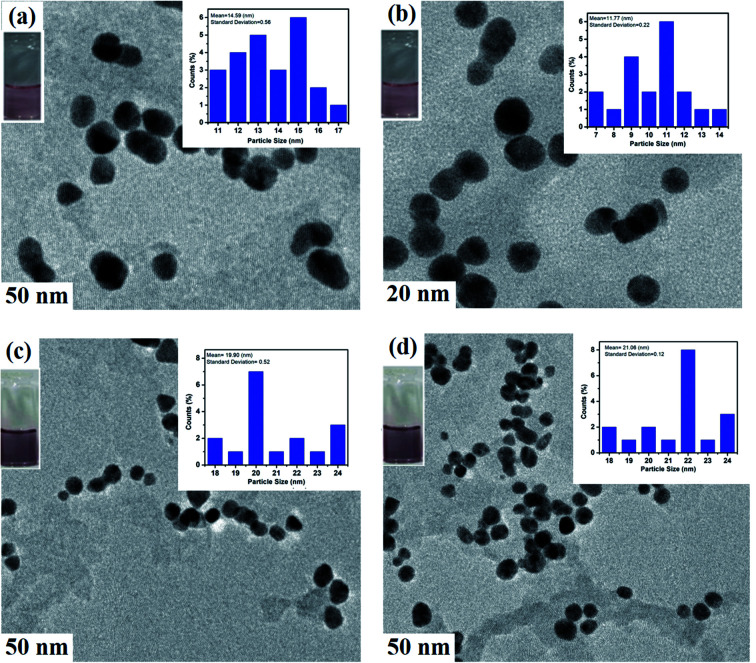
TEM images of (a) AuNPs, (b) IL–AuNPs, (c) IL–AuNPs–HSA and (d) IL–AuNPs–HSA with Glu.

IL–AuNPs and IL–AuNPs with HSA were characterized by TEM, DLS and FT-IR techniques. Fig. 2 shows the representative images of size distributions obtained for AuNPs, IL–AuNPs, IL–AuNPs with HSA and IL–AuNPs after the addition of Glu from TEM. This images shows that AuNPs shows more dispersity with IL in compare to bare-AuNPs because of high potential effect of IL upon AuNPs. It was observed that IL–AuNPs with HSA shows narrow size distribution and spherical shape by an increase in its average diameter (∼20 nm) due to electrostatic repulsion between the molecules, whereas, the sample solution was observed to be aggregated after the addition of Glu.^25,26^ The average diameter of the AuNPs, IL–AuNPs, IL–AuNPs with HSA and IL–AuNPs after the addition of Glu were ascertained to be 14.59 nm, 11.77 nm, 19.90 nm and 21.06 nm, respectively, which revealed successful modification of the surface of IL–AuNPs with HSA.

The hydrodynamic size of AuNPs, IL–AuNPs, IL–AuNPs with HSA and IL–AuNPs after the addition of Glu have also been revealed by DLS measurement, shown in Fig. S2,† where two peaks were observed for all the samples in the range of 1–10 nm and 10–100 nm. Out of both the peaks, the highest size distribution intensity was seen in the range of 10–100 nm for hydrated samples, thus we have considered them. The average size of AuNPs, IL–AuNPs, IL–AuNPs with HSA and IL–AuNPs after the addition of Glu was found to be 50 ± 3.7 nm, 20 ± 2.5 nm, 50 ± 2 nm and 50 ± 4.7 nm respectively. Here, size of the NPs increases from 20 to 50 nm when HSA was added into the NPs solution and aggregation was caused by the addition of Glu. These results are comparable to the size obtained from TEM imaging that indicates well-dispersivity of NPs with IL. The hydration state of NPs is the main reason for the difference caused in average diameter when characterized from TEM and DLS. DLS analysis was done using the hydrated samples of NPs whereas TEM image of the NPs is taken using the dried samples. Thus, there is an appreciable difference between the size evaluated using DLS and TEM techniques. However, TEM has been remarked as more significant for measuring the size of nanomaterials.^27^

The measurement of zeta potential indicates combination of electrostatic and hydrophobic interactions between the complex formed in sample solution. Almost all microscopic and macroscopic materials acquire an electronic charge on their surfaces in aqueous media. The zeta potential is an important indicator to identify charge on the surface of particles and used to predict stability of colloidal suspensions of NPs. Zeta potential measurements express a key role for understanding dispersion and aggregation processes of particles. A typical result obtained by the measurements of zeta potential is illustrated in Fig. S3,† which shows the effect of the addition of Glu and HSA on the NPs solution. Fig. S3(a) and (b)† shows the zeta potential of −19.3 mV and −14.2 mV for native HSA and IL–AuNPs, respectively. The surface potential of native HSA was found to be increased to −12.2 mV shown in Fig. S3(c),† after the addition of IL–AuNPs, which suggests the formation of IL–AuNPs–HSA complexes. The zeta potential values of the complex of IL–AuNPs–HSA was increased to −17.5 mV after the addition of Glu, Fig. S3(d).† This trend also indicates existence of electrostatic interactions between the protein and NPs.^28,29^ It was observed that increasing the surface charge on the particles also increases magnitude of interparticle electrostatic repulsion that gives confirmation about aggregation of sample solution.

**Fig. 3:**
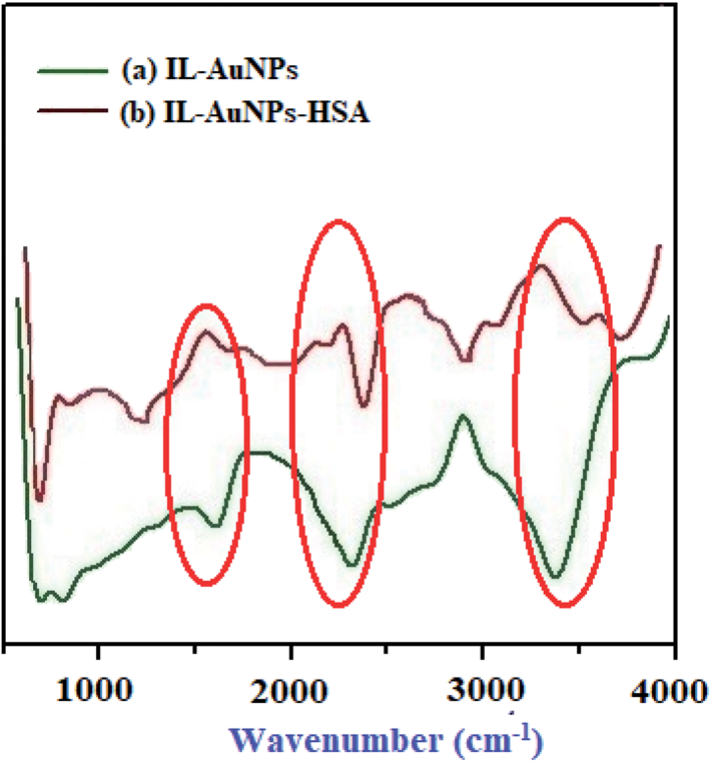
FT-IR spectra of IL–AuNPs and IL–AuNPs with HSA.

The binding of HSA with IL–AuNPs have been successfully identified by FT-IR measurements. Most of time, amino based IL is used as an alternative to complex with AuNPs. Fig. 3 shows the FT-IR spectrum of IL–AuNPs and IL–AuNPs with HSA. FT-IR spectra of IL–AuNPs consist of a strong and intense band due to stretching vibration at 3400 cm^−1^ (which is absent in IL–AuNPs–HSA), another band at 1690 cm^−1^ corresponding to amide group attributed to stretching vibration. The amino based IL plays an important role for determining their binding interaction to AuNPs. The strong stretching vibration band at 1690 cm^−1^ founds clearly in the IL–AuNPs which shows slightly shifts on coordination with AuNPs. This is strong evidence of surface binding of IL on the surface of AuNPs *via* amino linkage. The FT-IR spectra of IL–AuNPs shows a new band at 2300 cm^−1^ which was observed to be shifted to 2450 cm^−1^. A band appeared at 3000 cm^−1^ and 1400 cm^−1^ attributed to strong and weak carboxylic group stretching respectively which clearly indicates the participation of this group for strong electrostatic interaction.^30,31^

**Fig. 4:**
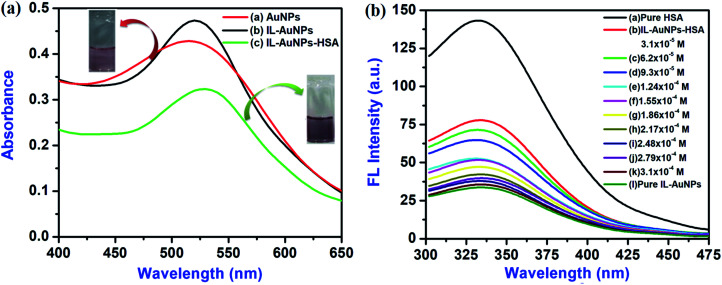
(a) Absorbance spectra of IL–AuNPs and IL–AuNPs with HSA and (b) Fluorescence emission spectra of IL–AuNPs with HSA at the concentration of 3.1 × 10^−5^ M to 3.1 × 10^−4^ M IL–AuNPs.

Subsequently, UV-Vis spectrum of pure-AuNPs gives broad spectra whereas IL–AuNPs gives sharp spectrum at 520 nm which was reduced after the addition of HSA, shown in Fig. 4(a). The digital picture of IL–AuNPs with and without HSA has also been inserted in Fig. 4(a). Here, pure IL–AuNPs solution shows red wine color but IL–AuNPs with HSA changes to purple color due to size enlargement and their aggregation behavior.

The fluorescence emission spectra of HSA at different concentration of IL–AuNPs (3.1 × 10^−5^ M to 3.1 × 10^−4^ M) were recorded in the wavelength range of 300–475 nm, shown in Fig. 4(b). Here, it was observed that as the concentration of IL–AuNPs increases, FL intensity of HSA decreases regularly by showing blue shifting of spectra. Hence, the highest emission wavelength was found at 338 nm and the lowest emission wavelength (after blue shift) was observed at 330 nm.

### 3.2 Fluorescence spectroscopic studies

#### 3.2.1 Quenching mechanism and effect of IL–AuNPs on HSA

**Fig. 5:**
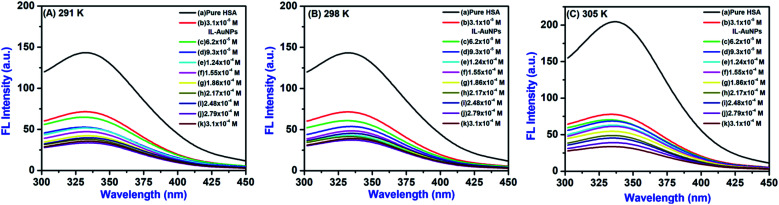
Fluorescence quenching spectra of HSA by IL–AuNPs at (A) 291 K, (B) 298 K and (C) 305 K.

Fluorescence quenching can be caused either by dynamic (collisions) or static (complex formation) with respect to the quencher.^15^ The quenching effect of IL–AuNPs on HSA was studied at three different temperature (*i.e.*, 291, 298 and 305 K), shown in Fig. 5. It could be seen that the FL intensity of HSA decreases regularly by increasing the concentration of IL–AuNPs and shows slightly blue shifting of spectra from 338 to 330 nm. This phenomenon implies that fluorescence quenching process was mainly controlled by dynamic quenching mechanism rather than a static quenching mechanism.^32^Table 1 also clarifies the dynamic method of this procedure by showing increase in the Stern–Volmer quenching constants (*K*_SV_) value upon increase in the temperature. The fluorescence quenching results were analyzed by the Stern–Volmer equation.^32–34^1
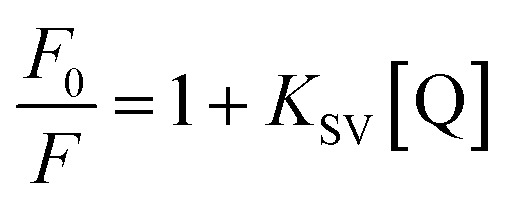
where, *F*_0_ and *F* are the fluorescence emission peak of HSA in the absence and presence of quencher, respectively. *K*_SV_ and [Q] are the Stern–Volmer quenching constant and the concentration of free quencher, respectively.

**Table 1:** Stern–Volmer quenching constants (*K*_SV_) and correlation coefficient (*R*) for IL–AuNPs with HSA at different temperatures

pH value	Temperature (K)	IL–AuNPs–HSA	
		*K*_SV_ (10^3^ L mol^−1^)	*R*
7.8	291	31.10 ± 0.05	0.996
	298	45.92 ± 0.08	0.987
	305	51.51 ± 0.06	0.984

**Fig. 6:**
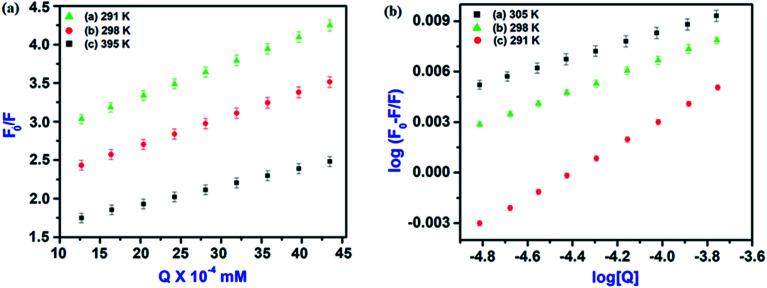
(a) Fluorescence quenching linear fit spectra of HSA by IL–AuNPs at different temperature (291, 298 and 305 K) and (b) Fluorescence quenching linear fit spectra at different temperature (291, 298 and 305 K).

The plot of *F*_0_/*F* for HSA *versus* IL–AuNPs concentration exhibits good linearity range (*R* ∼ 0.92) and affords *K*_SV_ to be 31 to 51 (10^3^ L mol^−1^) at the temperature of 291, 298 and 305 K, shown in Fig. 6(a). Therefore, it indicates that the fluorescence quenching process of HSA has been mainly governed by a dynamic quenching mechanism.

#### 3.2.2 The apparent binding constant (*K*_a_) and number of binding sites (*n*)

Several methods are available to calculate the binding constant (*K*_a_) and the number of binding sites (*n*).^35,36^ Here, HSA provides binding sites for IL–AuNPs. The reaction between IL functionalized AuNPs and HSA can be proposed as following:

2 Au + HSA → Au_1_–HSA

3 Au_1_–HSA + Au → Au_2_–HSA

4 Au_2_–HSA + Au → Au_3_–HSA

⋮

5 Au_*n*−1_–HSA + Au → Au_*n*_–HSA

where, HSA is the human serum albumin and Au is the colloidal, monodispersed gold nanoparticle functionalized with IL, Au_1_–HSA, Au_2_–HSA, Au_3_–HSA, …, Au_*n*−1_–HSA new complexes are formed showing the binding constant as *K*_a1_, *K*_a2_, *K*_a3_, …, *K*_a*n*_, respectively.

Based on this method, the obtained equation is:^27,32^6
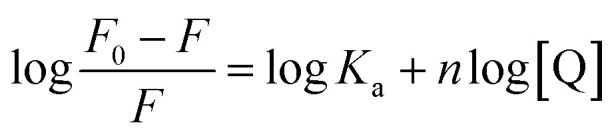
In the above equation, *F*_0_ and *F* are the fluorescence intensities of serum albumin in the absence and presence of quencher (IL–AuNPs), respectively. Here, *n* represents no. of binding sites, *K*_a_ is binding constant and *Q* is concentration of IL–AuNPs. Therein, the last linear fitting plots of log(*F*_0_ − *F*)/*F versus* log[Q] for the reaction of HSA and IL–AuNPs at different temperature are shown in Fig. 6(b). From the value of binding constant *K*_a_, it can be seen that the binding intensity of HSA to IL–AuNPs were increasing sharply upon the elevation of the temperature. Table 2 shows the calculated binding constant (*K*_a_) data of FL intensity which clarifies that binding constant increases more sharply at relatively higher temperature.

**Table 2:** Binding constants (*K*_a_) and correlation coefficient (*R*) for IL–AuNPs with HSA at different temperatures

pH value	Temperature (K)	IL–AuNPs–HSA	
		*K*_a_ (10^3^ L mol^−1^)	*R*
7.8	291	1.53 ± 0.09	0.995
	298	1.62 ± 0.07	0.991
	305	1.75 ± 0.11	0.986

### 3.3 Synchronous fluorescence study

**Fig. 7:**
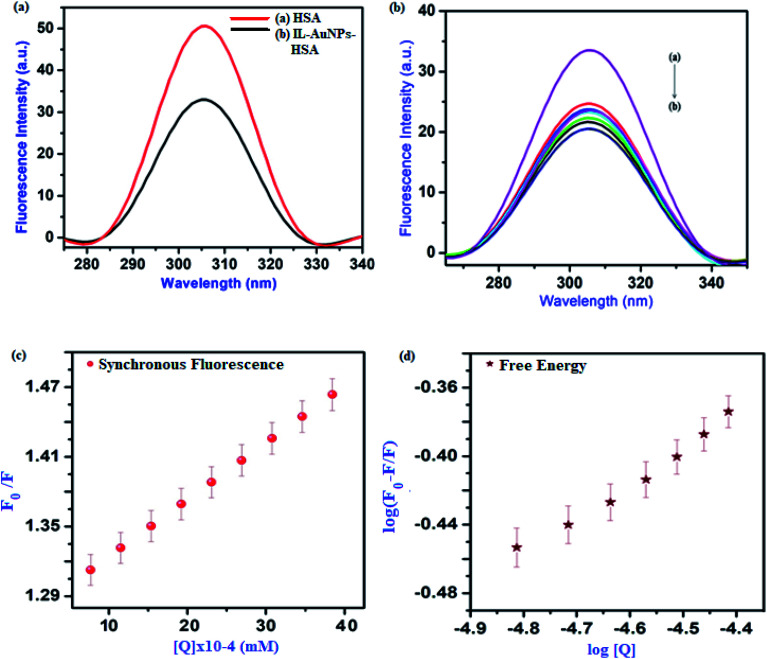
Synchronous fluorescence spectra, (a) quenching spectra of HSA by IL–AuNPs, (b) quenching effect on HSA at different concentration of IL–AuNPs from 3.1 × 10^−5^ M to 3.1 × 10^−4^ M, (c) synchronous fluorescence linear-fit spectra and (d) free-energy linear fit spectra.

Study of synchronous fluorescence spectra strongly gives a report for the molecular environment of the different functional group present in serum albumins.^37^ Spectra of synchronous are narrower and more symmetric than the fluorescence spectra, so the synchronous fluorescence method has been used as a conformational study.^38^ The synchronous fluorescence spectra obtained from IL–AuNPs with HSA are shown in Fig. 7(a). The surface modification of IL–AuNPs with HSA showed slight blue shift of the FL intensity peaks from 307 nm in Fig. 7(b), curve (a), to 304 nm, curve (b) which evidences that size of the inner IL–AuNPs “core” experiences a little decrease.^39^ Furthermore, the maximum FL intensity of HSA was significantly quenched with increasing quencher from 3.1 × 10^−5^ M to 3.1 × 10^−4^ M IL–AuNPs. As the concentration increases, the synchronous fluorescence spectral maximum gradually shifts to longer wavelengths and an explanation for this phenomenon can be given in terms of inner-filter effect.^40,41^ The linear fit and free energy plot has also been drawn for this method, shown in Fig. 7(c) and (d). Hence, the quenching effect of IL–AuNPs on the synchronous fluorescence emission of HSA was found to be dependent on the concentration of quencher.

Table 3 shows the calculated Stern–Volmer quenching constants (*K*_SV_), correlation coefficient (*R*) and binding constants (*K*) for IL–AuNPs with HSA system, using synchronous fluorescence quenching method at room temperature. This revealed that the conformation and microenvironment of HSA were changed by the binding of IL–AuNPs with HSA. From the dynamic ranges of IL–AuNPs–HSA at relatively higher temperature, it is very clear that the IL–AuNPs–HSA system provides more precise information about the molecular environments of HSA.

**Table 3:** Stern–Volmer quenching constants (*K*_SV_), correlation coefficient (*R*) and associative binding constants (*K*_a_) for IL–AuNPs with HSA at different temperatures

pH value	Temperature (K)	IL–AuNPs–HSA		
		*K*_SV_ (10^3^ L mol^−1^)	*R*	*K*_a_
7.8	291	7.012 ± 0.22	0.920	1.302
	298	8.002 ± 0.01	0.995	1.371
	305	8.930 ± 0.31	0.956	2.950

### 3.4 UV-Vis studies

**Fig. 8:**
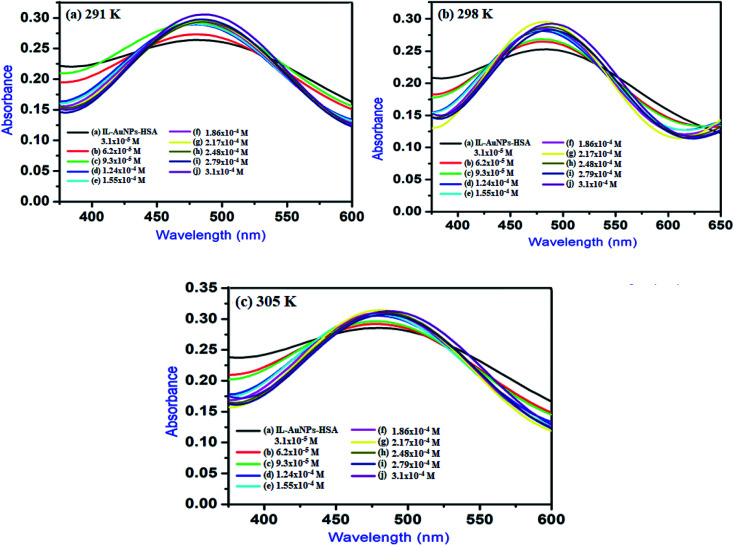
UV-Vis spectra of IL–AuNPs with HSA by increasing the concentration of IL–AuNPs from 3.1 × 10^−5^ to 3.1 × 10^−4^ at (a) 291 K, (b) 298 K and (c) 305 K.

Fluorescence quenching mechanism was further revealed by UV-Vis absorption spectrum for IL–AuNPs–HSA system. Fig. 8 shows the UV-Vis spectra of IL–AuNPs with HSA at the temperature of 291, 298 and 305 K. This study has been used for determination of binding constant (K) of IL–AuNPs–HSA complex using following equations.^32,33^7
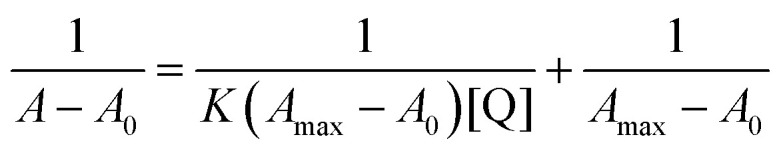
where, *A*_0_ and *A* are the absorbance of IL–AuNPs in the absence and presence of HSA, respectively. *A*_max_ are the absorbance at high concentrations of IL–AuNPs at saturation point and *K* is the binding constant. The plots of 1/[*A*_0_ − *A*] *versus* concentration of IL–AuNPs provides straight line. Fig. 8 shows the formation of complex of IL–AuNPs with HSA and calculated binding constant from Bensei–Hildebrand plot listed in Table 4.^42^

**Table 4:** Binding constant (*K*), correlation coefficient (*R*) and binding number (*n*) of the IL–AuNPs with HSA at different temperatures

pH value	Temperature (K)	IL–AuNPs–HSA		
		*K* ×10^4^	*R*	*n*
7.8	291	2.56 ± 0.03	0.998	0.0005
	298	2.60 ± 0.06	0.981	0.0083
	305	3.10 ± 0.04	0.994	0.0143

#### 3.4.1 Study of binding interaction between IL–AuNPs and HSA

In general, molecule binds with each other through any of four binding modes: hydrogen bonding, van der Waals force, electrostatic and hydrophobic interactions *etc.*^43^ As temperature grows up, binding interaction among IL–AuNPs and HSA increases sharply confirmed by calculation of binding constant (*K*) values, listed in Table 4. The thermodynamic parameters such as, change in enthalpy (Δ*H*), entropy (Δ*S*) and Gibb's free energy (Δ*G*) of reaction are important to confirm the acting force between IL–AuNPs and HSA. For this reason, binding constant (*K*) have been calculated at different temperature (291, 298 and 305 K), this is also because of HSA does not undergo structural degradation. The thermodynamic parameters can be calculated from the following equation:^32^8
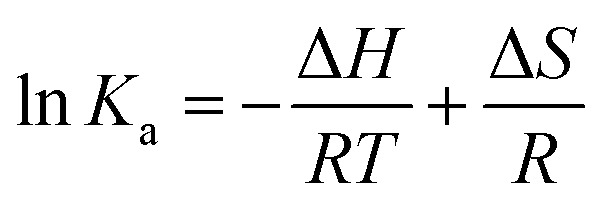

9 Δ*G* = Δ*H* − *T*Δ*S*

**Fig. 9:**
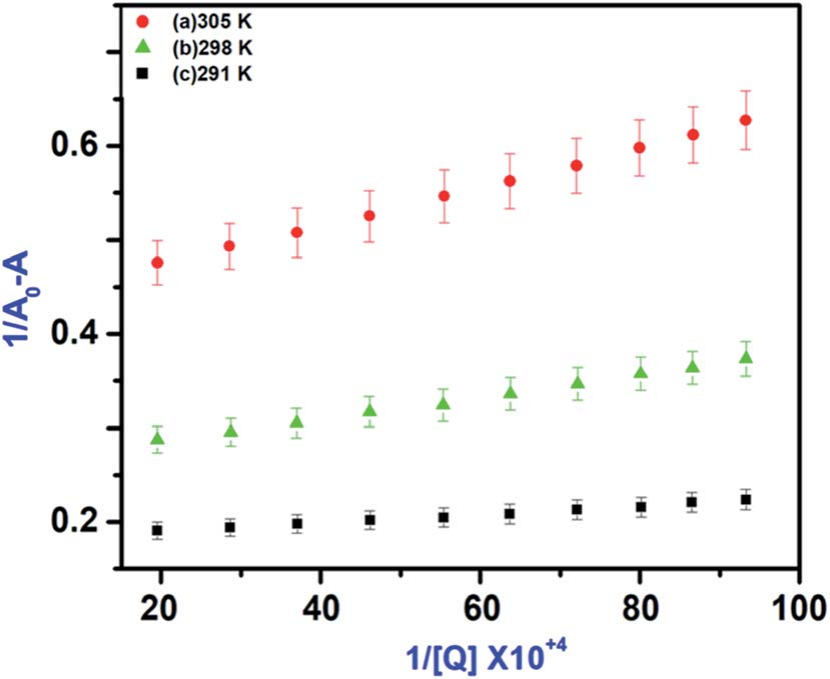
UV-Vis linear fit spectra of IL–AuNPs with HSA at different temperature (291, 298 and 305 K).

In these equations, *K*_a_ is the associative binding constant at temperature *T* and *R* is gas constant, respectively. Herein, Δ*H* shows energy released during the reaction process, Δ*S* for the hydrogen bonding interaction of IL–AuNPs with HSA and the negative sign of Δ*G* indicates the spontaneity of the reaction. The corresponding values of Δ*H*, Δ*S* and Δ*G* were determined from linear fit spectra obtained from UV-Vis studies of IL–AuNPs with HSA (Fig. 9). The values for these thermodynamic parameters are listed in Table 5. Several research work have been reported for the characteristic sign of the thermodynamic parameters related to interaction of IL–AuNPs with HSA.^14,15^ Reshma *et al.*^32^ showed binding of HSA to CDs and found binding constant 5.04 × 10^−4^ M, J. D. Berić *et al.*^44^ studied effect of metal ions on haloperidol and HSA and observed binding constant 7.94 × 10^3^ dm^−3^ mol^−1^. However, our system shows higher binding constant towards HSA and strong interaction with 2.6 × 10^4^ and strong binding with 3.10 × 10^4^. The binding of HSA to IL–AuNPs might involve electrostatic interactions. The negative sign of Gibb's free energy (Δ*G*) indicates that binding procedure of IL–AuNPs with HSA is spontaneous and negative value of enthalpy (Δ*H*) shows binding process as energy-releasing in nature.^45^

**Table 5:** Relative thermodynamic parameters (Δ*H*, Δ*S* and Δ*G*) for IL–AuNPs with HSA at different temperature

Temperature (K)	IL–AuNPs–HSA		
	Δ*H* (kJ mol^−1^)	Δ*S* (J mol^−1^ K^−1^)	Δ*G* (kJ mol^−1^)
291	−2.41 ± 0.05	192.58 ± 0.08	−56.46 ± 0.03
298	−2.47 ± 0.07	190.58 ± 0.06	−58.39 ± 0.01
305	−2.53 ± 0.04	191.00 ± 0.09	−60.60 ± 0.05

Serum albumins like BSA and HSA are able to bind a number of hydrophobic compounds among organic and inorganic materials.^43^ They are more stable and versatile to bind with other ligands, hence they are well-recognized principal component of blood plasma in comparison to all other proteins. HSA consists of three homologous fluorophores: phenylalanine, tryptophan and tyrosine generally named as domains (I, II and III).^46,47^ Among these sites, the main binding sites located in subdomains IIA and IIIA, such as tryptophan (Trp-214) and tyrosine (Tyr-411) residues.^48,49^ These residues are found in hydrophobic environment where Tyr residues such as Tyr-263, 319, 332, 334, 341, 353 and 370 are located in domain II of HSA and the location of Trp-214 is shown in Fig. S3.† HSA also shows binding ability to many of the long chain fatty acids (FAs) with their multiple binding sites, such as carboxylate moiety of fatty acids is anchored by electrostatic/polar interactions on the binding site of FA1-5. On the contrary, FA6 and FA7 have less affinity to bind with other ligands and hence it does not display a clear evidence of polar interactions.^50^ The fluorescence emission wavelengths of Tyr and Trp residues are found to be almost unchanged during the interaction which suggests the polarity around these residues is retained.^20^ In this method, HSA is found on the surface of IL functionalized AuNPs and are to be stable at relatively high temperature. This is initially confirmed by TEM and DLS and by calculation of different binding parameters. Then the selective competitive binding site of NPs in HSA can be carried out by two well known site markers warfarin and ibuprofen.^51,52^

### 3.5 Selective sensing of Glu using IL–AuNPs–HSA complex

**Fig. 10:**
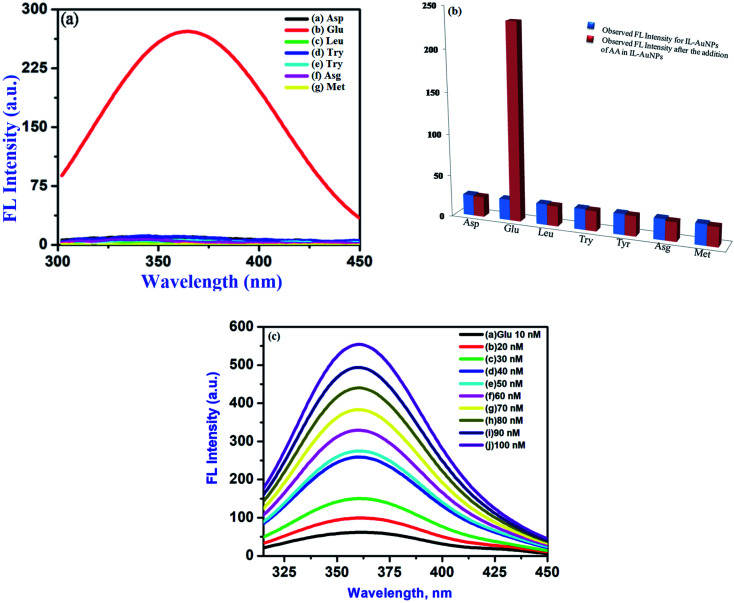
(a) Screening for selective detection of Glu amino acids (AA) using IL–AuNPs–HSA as a fluorescent probe for 10 min reaction time along with other AA, (b) FL intensity ratio for selectivity towards Glu and (c) effect of concentration of Glu (10–100 nM) on IL–AuNPs–HSA used as fluorescent probe for 3.1 × 10^−6^ M IL–AuNPs–HSA complex.

The practical applicability of this interactive study is to evaluate Glu in presence of other amino acids. Glu is most abundantly found amino acid in human blood and also used in the biosynthesis of proteins. This sensing phenomenon has been performed to find out the selectivity of AuNPs–HSA as fluorescent probe. For this, FL intensity curve for pure HSA, pure Glu, pure IL–AuNPs, IL–AuNPs–HSA and IL–AuNPs–HSA with Glu are shown in Fig. S5.† Herein, it could be seen that the Glu containing sample solution enhances the FL intensity more sharply at 360 nm, whereas, no effect has been observed for Asp, Leu, Try, Tyr, Asg and Meth with the same concentration 100 nM on FL signal, shown in Fig. 10(a). The FL intensity ratio observed by this selectivity procedure is shown in the Fig. 10(b). This process has been further optimized by the study on effect of concentration of Glu from 10–100 nM. Herein, we have found that with the increase in concentration of Glu, enhancement of spectra occurs, shown in Fig. 10(c). limit of detection (LOD) for this experiment was determined at three times of standard deviation (3 × *σ*) of blank divided by slope values. This system has also been compared with the result obtained using other techniques,^52–56^ shown in Table S1.† Herein, we observed that, our system gives lower LOD value 0.67 nM in the linearity range of 10–100 nM for Glu. Hence, this enhancement of spectra of IL–AuNPs–HSA due to Glu shows more selectivity and sensitivity with this approach. The performed method has also been study to find out the precision of this method by performance of repeatability of work. For this, it was observed that the enhancement of spectrum also appeared after a weak same as the freshly prepared sample. Further, the proposed methodology gives more précised value (1.45%) by calculating the percentage relative standard deviation (RSD%) for five successive analyses of Glu (10 nM) under the optimized condition. This gives confirmation about the feasibility and precision of the proposed sensing phenomena.

#### 3.5.1 Analytical performance of Glu

Statistically, analytical sensitivity has been provided to present the linear fit for Glu concentration on FL signals. For this, following linear correlation equation^57^ has been used

10 FL = 1.34 × *C* + 55.38

where FL is fluorescence intensity and *C* is the concentration of Glu.

**Fig. 11:**
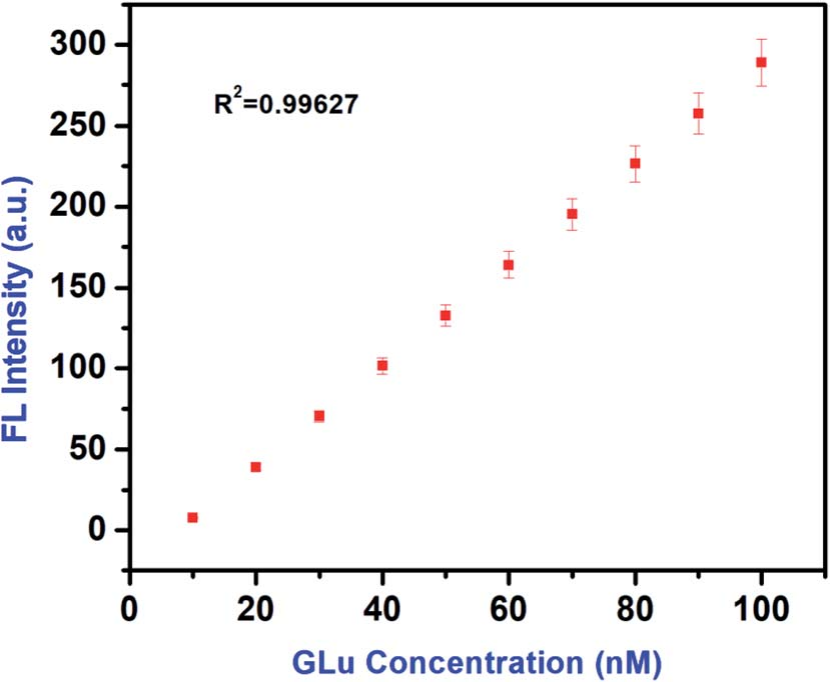
Plot of the calibration curve for Glu in the linear range of 10–100 nM.

A good linear correlation has been obtained by the use of eqn (10) between the concentration range of 10–100 nM of Glu and the FL signal intensity with a high correlation coefficient of *R*^2^ = 0.99627, shown in Fig. 11. This correlation curve also suggests that Glu gives a linear dose response assay. Herein, we report that Glu could achieve better analytical sensitivity for IL–AuNPs–HSA complex which is comparable to the sensitivity levels achieved with other conventional colorimetric assay (Table S1†).

### 3.6 Confirmational investigation by FT-IR spectroscopic studies

**Fig. 12:**
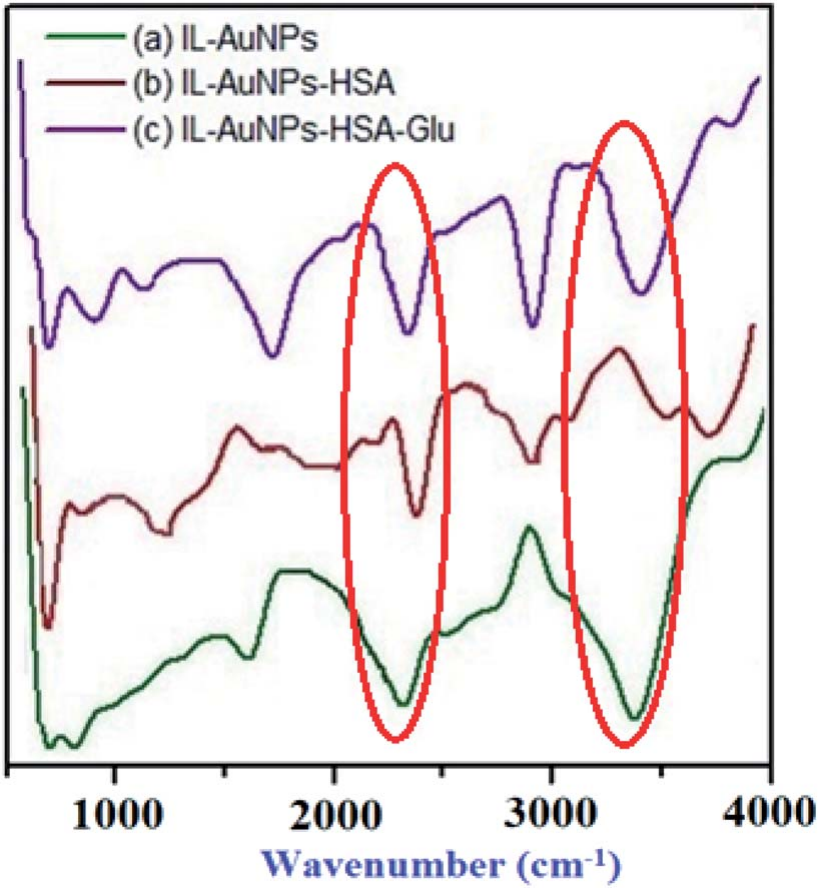
FT-IR spectra of IL-AuNPs, IL–AuNPs-HSA and IL–AuNPs–HSA with Glu.

The surface modification of IL–AuNPs in the absence and presence of HSA and with Glu has been confirmed by FT-IR measurement. The characteristic transmittance peaks (*ν*_as_(NH_2_) = 3350 cm^−1^) relates to the amino group found in IL–AuNPs have shifted to higher wavenumber 3650 cm^−1^, which indicates that HSA has successfully attached onto the surface of IL–AuNPs through the amino group of IL. One another broad peak of FT-IR spectrum was also observed at 3750 cm^−1^ for HSA. The FT-IR spectra also confirms the presence of amino and carboxylate ions in the AuNPs–Glu complex by reducing the peak observed for IL–AuNPs–HSA. This complex shows strong intense peak at 2900 cm^−1^ due to stretching of CH_2_. The peak at 1760 cm^−1^ revealed due to stretching of C=O from COOH group of Glu compound. The peak found at 1760 cm^−1^ for IL–AuNPs becomes more intense after the addition of Glu due to the binding of amino and carboxyl group onto the surface of AuNPs through the chemisorptions of carboxylate ions. It is perfectly ensured by the previous reported literature.^57,58^ Comparing the FT-IR spectra of IL–AuNPs, IL–AuNPs–HSA and AuNPs with Glu complex, we can find that IR-spectrum of IL–AuNPs resemble strongly with AuNPs–Glu complex that of IL–AuNPs–HSA, as shown in Fig. 12.

## 4. Conclusions

We have successfully demonstrated highly efficient IL–AuNPs–HSA as fluorescent probe for selective detection of Glu in presence of other amino acids. The amino based IL was used as stabilizing agent to modify the surface of AuNPs which has also helped in reducing self-induced aggregation of AuNPs ultimately making them mono-dispersive in nature. In present investigation, the interaction between IL–AuNPs with HSA has been investigated at three different temperatures which reveals that stronger binding was found at the highest temperature. The fluorescence technique indicated that the quenching mechanism was be dynamic and proves to be a selective platform for sensing of Glu. The size of IL–AuNPs–HSA was evaluated by TEM and DLS. The interaction parameters were evaluated by employing fluorescence, synchronous fluorescence and UV-Vis techniques. This binding process was then applied for selective detection of Glu with LOD value of 0.67 nM. Thus, the method found to be feasible and reproducible for sensing of bioactive compounds in nearby future. Moreover, these amino based IL–AuNPs is attributed with more biocompatibility, least toxicity which could be further employed as highly sensitive bio-sensing probe.

## Conflicts of interest

There are no conflicts to declare.

## Supplementary Material

RA-010-D0RA04394J-s001
