# Supplementary material for: Thermodynamic investigation of the interaction between ionic liquid functionalized gold nanoparticles and human serum albumin for selective determination of glutamine
Source: RSC Adv. 2020 Aug 26;10(52):31400–10. doi: 10.1039/d0ra04394j (PMC9056378; doi:10.1039/d0ra04394j)
Supplement: RA-010-D0RA04394J-s001 [file RA-010-D0RA04394J-s001.pdf]

Electronic Supplementary Material (ESI) for RSC Advances.
This journal is © The Royal Society of Chemistry 2020

Thermodynamic Investigation of the Interaction between Ionic Liquid Functionalized Gold Nanoparticles and Human Serum Albumin for Selective Determination of Glutamine

Sushama Sahu,[a] Reshma,[a] Srishti Sharma,[a] Indrapal Karbhal[a] and Kallol K. Ghosh*[a]

[a]School of Studies in Chemistry, Pt. Ravishankar Shukla University, Raipur-492010 (C.G.), India

SUPPLEMENTARY MATERIALS

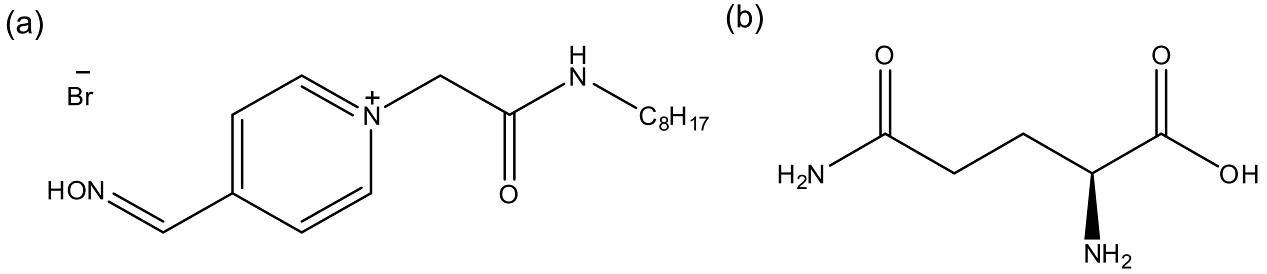

Fig. S1 Chemical structure of (a) amino based ionic liquid [4-((hydroxiimino)methyl)-1-(2-(octylamino)-2-oxoethyl)pyridine-1-iumbromide] and (b) glutamine.

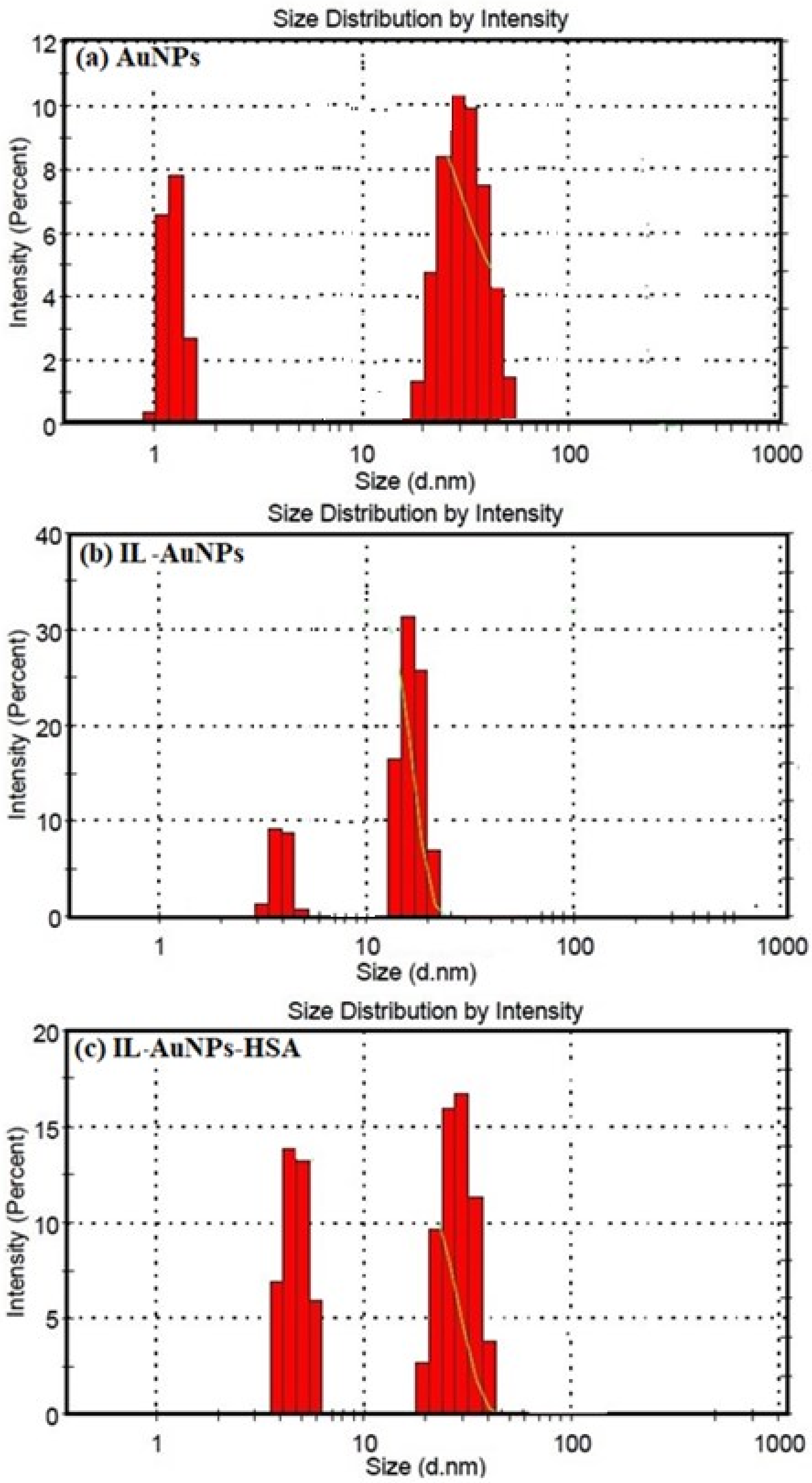
Size Distribution by Intensity
(a) AuNPs
Intensity (Percent)
Size (d.nm)
Size Distribution by Intensity
(b) IL -AuNPs
Intensity (Percent)
Size (d.nm)
Size Distribution by Intensity
(c) IL-AuNPs-HSA
Intensity (Percent)
Size (d.nm)

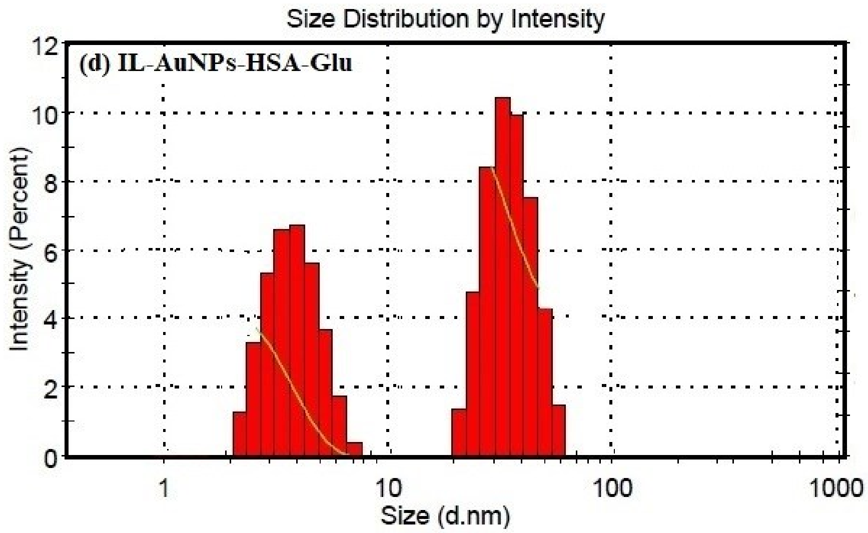

Fig. S2 DLS size distribution plots of (a) AuNPs, (b) IL-AuNPs (c) IL-AuNPs-HSA and (d) IL-AuNPs-HSA with Glu.

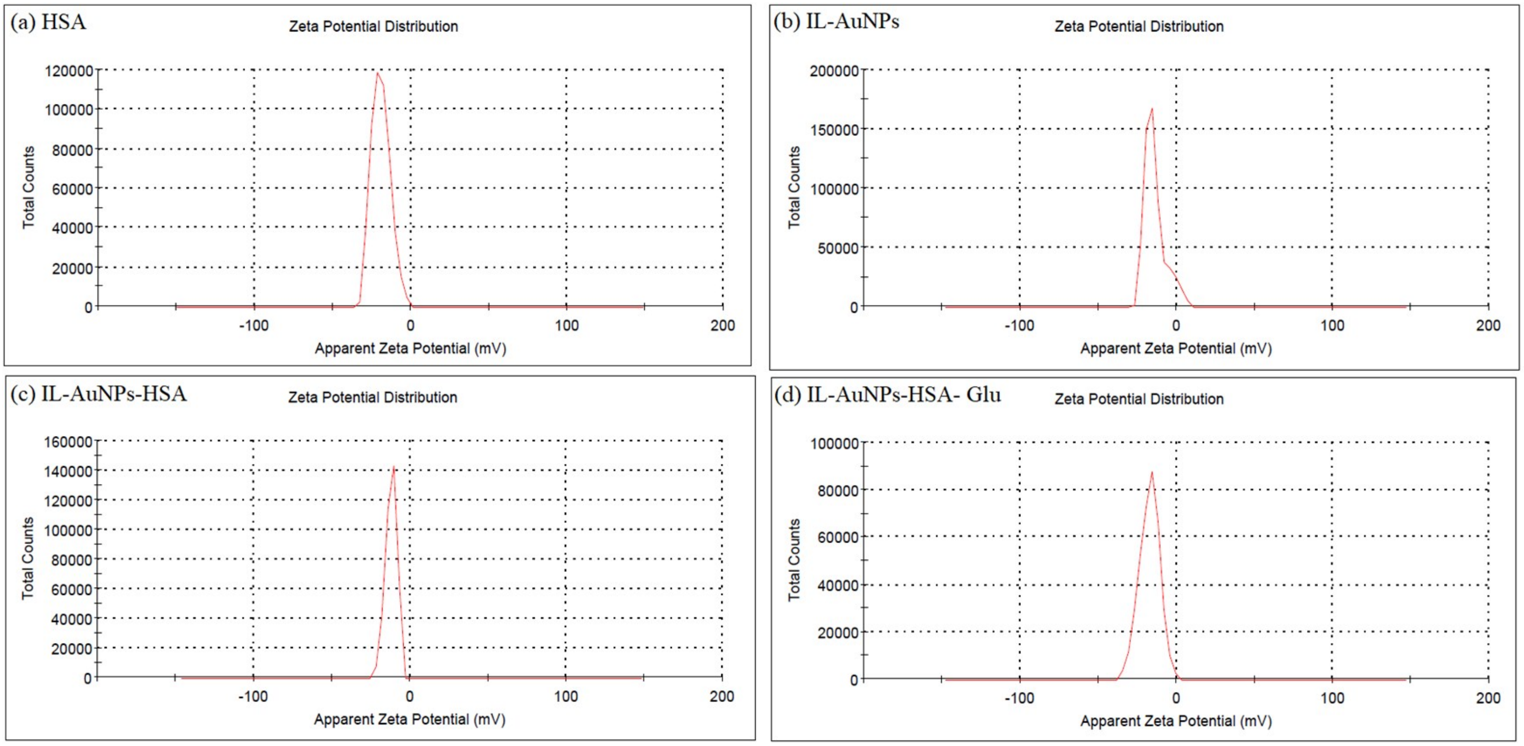

Fig. S3 Zeta potential measurements of (a) HSA, (b) IL-AuNPs (c) IL-AuNPs-HSA and (c) IL-AuNPs-HSA with Glu.

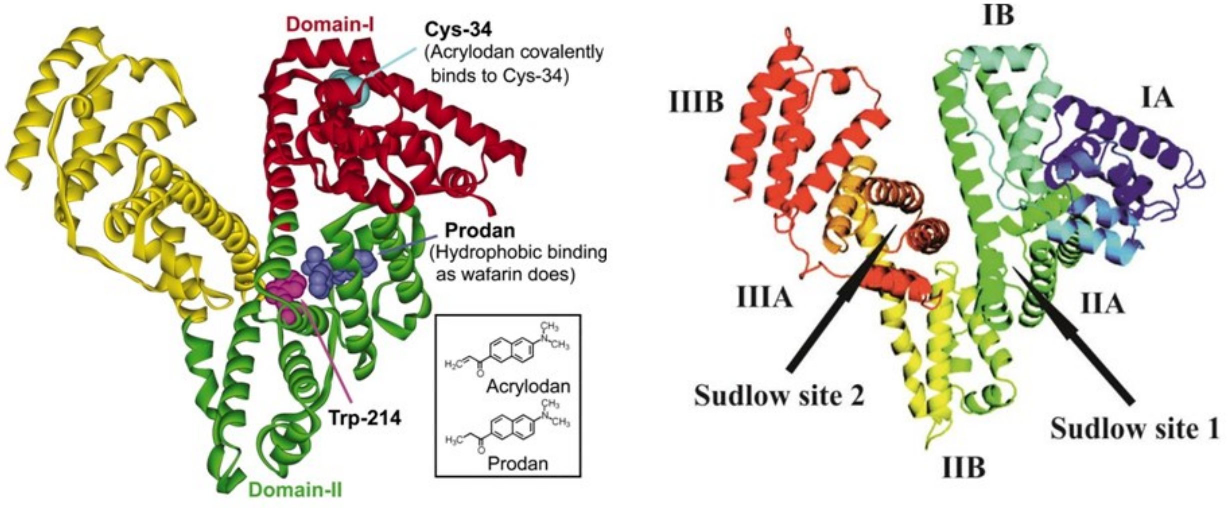

Fig. S4 Structure of HSA showing three different domains.

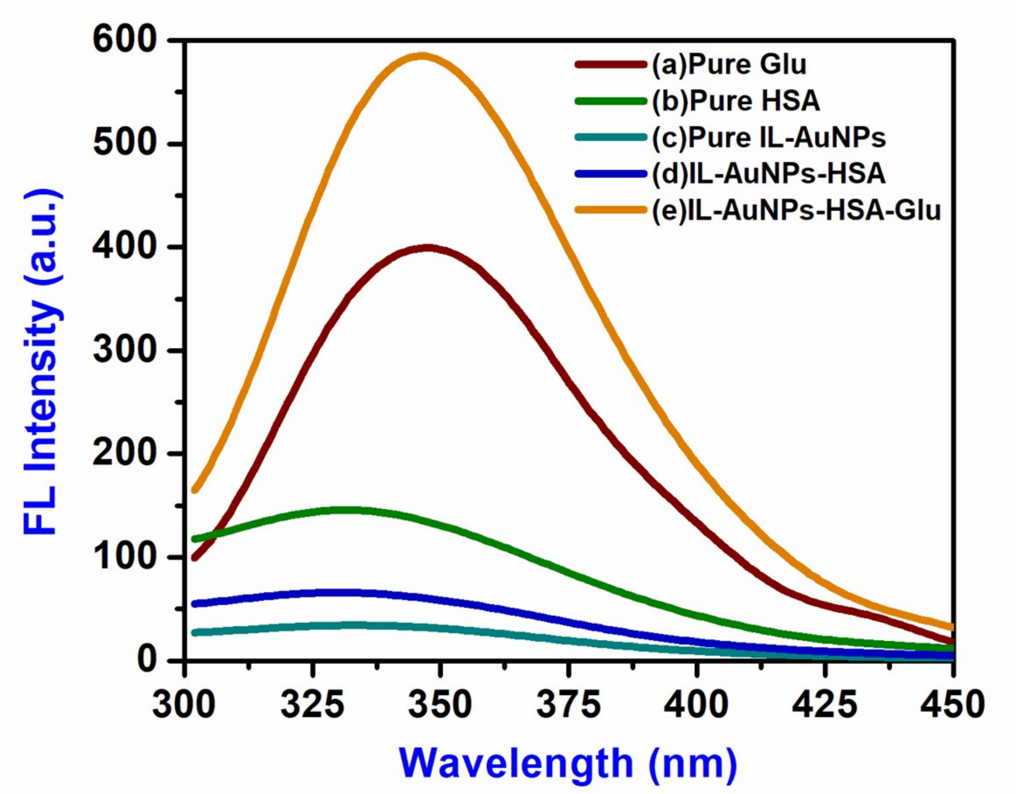

Fig. S5 Fluorescence spectra for (a) pure Glu, (b) pure HSA, (c) pure IL-AuNPs, (d) IL-AuNPs-HSA and (e) IL-AuNPs-HSA with Glu.

Table S1. Comparison of sensitivities of detection techniques with the proposed methodology

Method	Analyte	Concentration	Correlation Coefficient	Limit of Detection	Ref
Colorimetry	Lysozyme	100-1000 nM	0.9890	50 nM	52
Colorimetry	Lysozyme	20 nM to 2 μM	0.99	20 nM	53
Impedimetric	MWCNT-SPEs-aptamer	0-28.6 μM	0.9984	862 nM	54
Capillary Electrophoresis	Lysozyme	0.005-0.3 μM	0.9930	8 nM	55
Fluorescence	FITC-BSA	0.0-0.10 μg/ml	0.94		56

Colorimetry	Glutamine	10-100 nM	0.99627	0.67	Present Method
